# Antimicrobial susceptibility against metronidazole and carbapenem in clinical anaerobic isolates from Pakistan

**DOI:** 10.1186/s13756-019-0549-8

**Published:** 2019-06-14

**Authors:** Yusra Shafquat, Kauser Jabeen, Joveria Farooqi, Kiran Mehmood, Seema Irfan, Rumina Hasan, Afia Zafar

**Affiliations:** Department of Pathology and Laboratory Medicine, Aga Khan University Hospital, Stadium road, Karachi, Pakistan

**Keywords:** Anaerobic antimicrobial susceptibility, Anaerobic resistance to metronidazole, Anaerobic resistance to carbapenem, Risk factors associated with resistance

## Abstract

**Background:**

Globally metronidazole and carbapenem resistance in anaerobic organisms is increasing necessitating continuous surveillance to guide selection of empirical treatment. In this study we have determined metronidazole resistance in anaerobes using MIC Evaluator strips (M.I.C.E strips). Carbapenem resistance was evaluated only in metronidazole resistant isolates.

**Material and methods:**

The study was conducted at the Aga Khan University (AKU) Hospital laboratory, Karachi, Pakistan (2014–2017). Metronidazole and imipenem resistance was evaluated using M.I.C.E strips and minimum inhibitory concentrations (MICs) were interpreted using Clinical Laboratory Standards Institute (CLSI) criteria. Clinical details including demographics, prolonged hospital stay, malignancy, transplant, dialysis, diabetes, site of infection and outcome were analyzed for association with metronidazole resistance.

**Results:**

Of the 223 clinically significant isolates, 39 (17.5%) were metronidazole resistant (excluding the inherently resistant organisms; for example *Cutibacterium* species). Imipenem resistance was determined in 29 metronidazole resistant isolates and of these 7 (24.1%) were found to be resistant. Proportion of metronidazole resistant strains was highest amongst *Bacteroides* species. A significant increase in metronidazole resistance from 12.3% in 2010–2011 to 17.5% in the current study was found. Carbapenem resistance also emerged in the period 2014–2017.

Isolates from malignancy and transplant patients showed lower odds of developing metronidazole resistance (0.003(95% CI: 1.7–17.9)). Prolonged hospital stay was not associated with metronidazole resistance (1.1((95% CI: 0.5–2.5)).

**Conclusion:**

The rising trend of metronidazole resistance and emergence of carbapenem resistance in anaerobic bacteria is alarming. Continued surveillance with strengthening of laboratory capacity regarding anaerobic susceptibility testing is urgently needed in Pakistan.

## Background

Anaerobic bacteria are known to be associated with a number of human infections [1] including intra-abdominal, genital tract, surgical site, brain abscesses and skin and soft tissue infections [2]. Metronidazole is an important first-line anti-anaerobic agent, but also frequently used for community based infections such as ameobiasis, giardiasis, trichomoniasis, bacterial vaginosis and *Helicobacter pylori* associated gastritis and peptic ulcer disease [3–5]. Studies show that the resistance to metronidazole and imipenem in anaerobic organism varies globally. However, emergence of strains with combined resistance to metronidazole and carbapenem hampers treatment and is associated with poorer outcomes [6, 7]. Guidelines therefore highlight the increasing importance of susceptibility testing in individual settings [8, 9] .

In countries with limited laboratory capacity, isolation and susceptibility testing of anaerobic bacteria is usually not performed. Therefore, treatment is mostly empirical and if appropriate anti-anaerobic agents are not used, treatment outcome could be affected. Hence, in such setting there is a need for updated geographical and clinically relevant data [10]. In addition to monitoring trends of antimicrobial resistance, routine susceptibility testing of anaerobic organisms isolated from blood and sterile body sites is recommended for clinical laboratories [10, 11].

An earlier study from Pakistan conducted by same study group during 2010–2011 had reported metronidazole resistance rate of 12.3% amongst anaerobic isolates [12]. Metronidazole is one of the highly prescribed agents in the country and as per published reports its empirical use is 23.4% in cases of community acquired gastroenteritis in the country to cover parasitic etiology [5, 13]. The fact that metronidazole is easily available “over the counter” contributes to its frequent use and thus to the rising resistance trend [14]. Additionally, data also shows that sales of carbapenem is highest in Pakistan and India amongst developing countries [15]. In clinical practice, carbapenems are used as a second line option especially in hospital settings. Although carbapenem resistance in anaerobic organisms was not reported from Pakistan previously, its status currently needs evaluation especially in metronidazole resistance strains. Therefore, in this study we aimed to determine to metronidazole resistance in anaerobic organisms isolated at the Aga Khan University Hospital (AKUH) Clinical Laboratory. Additionally, carbapenem resistance was also determined in metronidazole resistant strains. Association between metronidazole resistance, and prolonged hospital stay and patient outcome was also determined.

## Material and methods

**Fig. 1:**
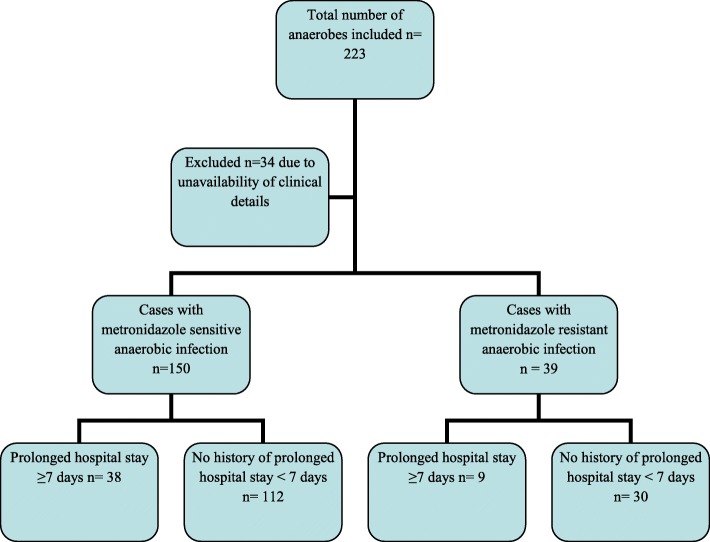
Flow chart showing the study design and distribution of study population on the basis of prolonged hospital stay (primary exposure)

The cross-sectional study was conducted at the microbiology section of the AKUH clinical laboratory in Karachi, Pakistan. AKU hospital is a tertiary care hospital. Clinical laboratory receives specimens from admitted patients as well as outpatients who deposit specimens at more than 200 collection points located in major cities and towns of Pakistan. Consecutive sampling technique was applied and all clinically significant anaerobes isolated from blood, sterile body fluids, pus, tissue and bone cultures from January 2014 to August 2017 were included. Isolates identified as colonizers on the basis of history, source and clinical data, such as localized perianal abscess, were not included. Duplicate isolates from the same patient isolated from more than one specimen, during the same infectious episode or hospital admission, were excluded (Fig. 1). The study was exempted from ethical approval by the Institutional Ethical Review Committee (4068-pat-ERC-16).

### Clinical data

Clinical details were collected as part of laboratory reporting protocol and included demographics, prolonged hospital stay, malignancy, transplant, dialysis, diabetes, site of infection and outcome, which were further analyzed for association with metronidazole resistance. Due to non-availability of data on prior antibiotic usage, prolonged hospital stay was taken as a surrogate for extensive antimicrobial use. We hypothesized that the odds of metronidazole resistance will be higher in patients with prolonged hospital stay as increased length of hospital stay is associated with increased antibiotic use. Clinically significant isolates were defined as those isolates that were isolated from either sterile sites or non-sterile sites with symptoms and signs consistent with anaerobic infection and require targeted therapy. Prolonged hospital stay was defined as a hospital stay of ≥7 days.

### Isolation and identification of anaerobic bacteria

Anaerobic organisms were isolated directly from clinical specimens as well as on subculture of the cooked meat broth inoculated for enrichment for 24 h. The sample or broth was inoculated on sheep blood agar with 50 μg diagnostic metronidazole disc (Oxoid). The culture plates were incubated in an anaerobic chamber (Concept plus RUSKINN) for 48 h at 36 ± 1 °C. The anaerobic organisms were identified by gram stain, colony morphology, aerotolerance, esculin hydrolysis in the presence of bile, and API® 20A system (bioMerieux®, Marcy l’Etoile, France).

### Susceptibility testing

Metronidazole MIC evaluator strips (M.I.C.E), (Oxoid™, Thermo scientific™, Basingstoke, Hants, UK) were used to determine MICs according to the manufacturer’s instructions. CLSI recommended Brucella agar supplemented with Hemin, Vitamin K1 and 5% pooled sheep blood was used for susceptibility testing. The MICs were read after 24 h and, in cases where it was difficult to interpret, at 48 h. The MICs were interpreted as sensitive (≤8 μg/ml) and resistant (≥16 μg/ml) using CLSI breakpoints [16]. The MIC range, MIC_50_ and MIC_90_ were determined for metronidazole. *Bacteroides fragilis* ATCC 25285 was used as control. Clinical isolates were saved at − 80 °C in glycerol phosphate broth and metronidazole resistant strains were revived to perform imipenem susceptibilities. Due to fastidious nature of the organisms, all the metronidazole resistant strains could not be recovered and imipenem susceptibilities could only be determined using M.I.C.E strips (Oxoid™, Thermo scientific™, Basingstoke, Hants, UK) against 29 metronidazole resistant isolates.

### Statistical analysis

The data obtained were entered into the statistical software SPSS version 19.0 (SPSS, Inc., Chicago, IL). For descriptive analysis, mean and standard deviation of continuous variables such as age and MICs were computed. For categorical variables, e.g. gender and antibiotic resistance, frequencies and percentages were calculated. Association of risk factors with metronidazole resistance was determined using binomial logistic regression to obtain odds ratios with 95% confidence intervals (95%CI). A *P*-value less than 0.05 was considered significant on univariate and multivariate analysis.

## Results

A total of 223 anaerobes were isolated from January 2014 to August 2017. Out of which 146 strains were obtained from male (65.4%) and 77 from female patients (34.5%). Isolates from patients aged < 18 year were 41/223 (18.3%), 18–60 years were 146/223 (65.4%) and > 60 years were 36/223 (16%). Isolation of organisms varied geographically with 78.5% (175/223) of the samples from Sindh, 9% (20/223) from Punjab, 9% (20/223) from Balochistan and 3.6% (8/223) from Khyber Pakhtunkhwa. Organisms were most commonly isolated from patients with intra-abdominal infections (*n* = 66), bacteremia (*n* = 41), brain abscess (*n* = 12), necrotizing fasciitis and osteomyelitis (*n* = 19), diabetic foot (*n* = 11), surgical site infections (*n* = 10), perianal fistula and ischiorectal abscess (n = 12), ovarian and uterine abscesses (n = 11), gluteal abscess (*n* = 7), empyema lung (n = 6),,necrotizing otitis media (n = 1), abscesses from other body sites and other pus samples (*n* = 27). Fifty-eight percent of the isolates were from poly-microbial infections. Clinical information was available for 189/223 (85%) of the patients. Of the 189 cases in which outcome could be assessed, the case fatality rate was 5.2% (10/189). Most commonly isolated organisms were *Bacteroides* species (77%) followed by *Clostridium* species (18%).

### Antimicrobial susceptibility data

Metronidazole resistance was detected amongst 39/223 (17.5%) isolates (Table 1). Resistance was mainly seen in *Bacteroides* species (16%). MIC ranged from < 0.015 to > 256 μg/ml and MIC_50_ and MIC_90_ was 2 μg/ml and > 256 μg/ml respectively*.* Seven (24.1%) of the 29 metronidazole resistant strains tested for imipenem susceptibilities were found to be resistant. Of the 7 metronidazole sensitive strains tested for imipenem susceptibilities, resistance was detected in only one (14.2%). Organisms inherently resistant to metronidazole were excluded from the comparison of metronidazole resistance in both study periods. An increasing trend of resistance was noted in metronidazole, as 12.4% tested strains were metronidazole resistant in 2010–2011 [12] compared to 17.5% in current study but the rise was found to be of no statistical significance (Table 1). Results were similar when assessed individually amongst the species.

**Table 1:** Trends in antimicrobial resistance of anaerobes in Pakistan from 2014 to 2017 compared with 2010–2011 [12]

Organisms	*n* = 223	Metronidazole resistance (2014–2017) n (%)	Metronidazole resistance (2010–2011) n (%)	*P* value (Pearson chi square)	Frequency of isolates tested for imipenem (*n* = 29) n (%)	Imipenem resistance (*n* = 7) n (%)	Imipenem resistance (2010–2011) *N* = 0/106
Overall resistance (excluding inherent resistance)		39/223 (17.4))	13/106 (12.3)	0.184	–	7/29 (24.1)	0/106
*Bacteroides fragilis*	97	20 (20.6)	6/39 (15.3)	0.483	21	6	0
*Bacteroides* species	74	16 (21.6)	06/28(18)	0.983	5	1(27)	0
*Clostridium* species	39	2 (5)	1/32 (3)	0.676	3	0	0
*Fusobacterium* species^a^	3	0	0/1 (0)	–	–	–	–
*Prevotella* species^a^	3	1 (33)	0/6 (0)	–	–	–	0
*Cutibacterium* species	5	5^b^	–	–	–	–	–
*Bifidobacterium* species^a^	1	1^b^	–		–	–	–
*Eggerthella lenta* ^a^	1	1^b^	–		–	–	–

Species marked (^a^) were included in “Other” category in regression analysis for association with drug resistance

^b^ Species inherently resistant to metronidazole - Organisms inherently resistant to metronidazole were not included in the comparison of metronidazole resistance between the two study periods

### Risk factors

The frequencies of risk factors assessed during this study are shown in Table 2; Table 3 displays the details of regression analysis. Logistic regression was performed to better understand which groups are at greater risk of infections with metronidazole resistant strains, whether acquired or inherent. The data obtained can be used to guide physicians and surgeons about appropriate empirical therapy in various infections. Amongst the anaerobes studied, the odds of resistance were highest in *Bacteroides* species, though not statistically significant (1.2 (95% CI: 0.5–3.0)). The chance of isolating metronidazole resistant anaerobes from patients with malignancy and transplant was low (*5.6* (95% CI: 1.7–17.9)). Prolonged hospital stay was not significantly associated with metronidazole resistance (1.1 ((95% CI: 0.5–2.5)).

**Table 2:** Overall frequency of risk factors and amongst metronidazole and imipenem resistant strains

Risk factors	Total (189)	Metronidazole resistance (total = 33)	Imipenem resistance (total = 26 metronidazole resistant strains)
	N (%)	N (%)	N (%)
Age			
< 18 years	31 (16.4)	3 (9.09)	1 (3.8)
18–60 years	126 (66.7)	24 (72.7)	3 (11.5)
> 60 years	32 (16.9)	6 (18.2)	3 (11.5)
Gender			
Male	129 (68.2)	26 (78.8)	7 (26.9)
Female	60 (31.7)	7 (21.2)	0
Prolonged hospital stay^a^	43 (22.7)	9 (27.3)	3 (11.5)
Diabetes Mellitus	26 (13.7)	2 (6.06)	0
Malignancy/Transplant	11 (5.82)	5 (15.1)	1 (3.8)
Dialysis	2 (1.06)	1 (3.03)	0
Bacteremia	35 (18.5)	9 (27.3)	3 (11.5)
Skin and soft tissue infections	49 (25.9)	6 (18.2)	1 (3.8)
Head and neck infections	18 (9.52)	1 (3.03)	0
Intra-abdominal infections	66 (34.9)	13 (39.4)	2 (7.7)
Empyema lung	6 (3.17)	2 (6.06)	1 (3.8)
Genitourinary tract infections	17 (8.99)	2 (6.06)	0
Expired	10 (5.29)	3 (9.09)	3 (11.5)

^a^Primary exposure

**Table 3:** Univariate and multivariate analysis of the risk factors identified. All those with statistically significant Odds Ratios, at 95% confidence, are in bold

	Metronidazole resistance as outcome (*n* = 189)			
	Univariate analysis		Multivariate analysis	
Clinical characteristics	OR (95% CI)	*P* Value	OR(95% CI)	*P* Value
Prolonged hospital stay^b^ (≥ 7 days of hospital admission)	1.1 (0.5–2.5)	0.869	0.6 (0.2–1.7)	0.381
Age group (< 18 years as reference)		0.338		
18–60 years	2.6 (0.7–9.0)	0.144		
> 60 years	2.5 (0.6–10.7)	0.214		
Female (Male as reference)	0.5 (0.2–1.2)	0.128		
Organisms (Clostridium species as reference)		0.876		
*Bacteroides fragilis*	0.6 (0.06–5.1)	0.611		
*Bacteroides* other than fragilis	1.2 (0.5–3.0)	0.662		
Others^a^	1.016 (0.3–3.1)	0.978		
Diabetes Mellitus	0.3 (0.06–1.3)	0.119		
Malignancy/transplant	5.6 (1.7–17.9)	**0.003**	6.3(1.7–23.3)	**0.005**
Dialysis	4.2(0.2–68.5)	0.315		
Bacteremia	2.3 (1.0–5.1)	**0.041**	2.1 (0.9–4.7)	**0.089**
Skin and soft tissue infection	0.6 (0.2–1.3)	0.181		
Head and neck infections	0.4 (0.09–2.0)	0.302		
Intraabdominal infections	1.1 (0.5–2.4)	0.739		
Respiratory tract infections	2.1 (0.4–11.9)	0.399		
Genitourinary infections	0.5 (0.1–2.4)	0.402		
Expired	2.5 (0.7–9.0)	0.161		

^a^ “Other” category includes *Fusobacterium* species, *Cutibacterium* species, *Prevotella, Bifidobacterium, Eggerthella lenta*

^b^primary exposure

## Discussion

A high metronidazole resistance rate (17.5%) is noted in the current study. Emergence of carbapenem resistance in anaerobic organisms has also been reported for the first time in this study. Resistance was most frequently seen in *Bacteroides* species which was also the most commonly isolated organism. There is also an increasing trend of metronidazole resistance (12.4% in 2010–2011 to 17.5% in 2014–2017) [12]. Previous study conducted by the same group assessing resistance in clinical anaerobic isolates collected from AKUH laboratory using similar sampling strategy had reported 12.4% metronidazole resistance. The susceptibility method used in the previous study was CLSI recommended agar dilution, while this study has been conducted using comparable methodology i.e. exponential gradient method. The findings of both studies reflect the rising trend of antimicrobial resistance in anaerobes in Pakistan and necessitate the need of routine susceptibility testing across laboratories in Pakistan. However, there are several limitations in establishing anaerobic susceptibility testing in the country. Firstly, isolation of anaerobes requires special environment and media, which is not available in many diagnostic laboratories. In addition, disc diffusion due to its ease and low cost is the most commonly used method of susceptibility testing. Disc diffusion technique for the susceptibility testing of anaerobes has not been recommended by both CLSI and EUCAST, although a study has shown good agreement between disk diffusion and agar dilution method in *Bacteroides* species for metronidazole and imipenem [17]. Agar dilution for anaerobe susceptibility requires expertise and is not feasible for most local laboratories. Use of M.I.C.E strips and E-test have good correlation with agar dilution and are reliable, but are expensive for laboratories with limited resources [18, 19]. Hence we suggest investment in financial terms and capacity building of staff in both public and private sector laboratories across Pakistan to promote accurate reporting of susceptibility testing.

Multiple centers worldwide have reported increasing metronidazole resistance in anaerobes, mainly in *Bacteroides* species. In a meta-analysis conducted in France over an interval of 11 years, a 2.8 fold rise in metronidazole resistance was observed in *Bacteroides* and *Parabacteroides* species [10]. Similarly in other studies conducted in Ontario, Canada, South Africa and Croatia, India, 1.2, 8, 2.9 and 24.5% of the *Bacteroides* species were found to be resistant to metronidazole respectively [1, 20–22]. In another study from Netherlands, 4% of the *Prevotella* species were found resistant to metronidazole [11]. A comparative analysis of 11 year data of anaerobe antimicrobial susceptibility conducted in Kuwait demonstrated metronidazole resistance rate to be as high as 2.7% in *Bacteroides fragilis* and 5.6% in *Bacteroides ovatus* respectively [2]. Global emergence of metronidazole resistance has led to use of carbapenem for the treatment of complicated intra-abdominal infections. A study conducted in Taiwan, over a period of 16 years, reported imipenem and meropenem resistance in 7 and 12% of *B. fragilis* isolates, 4 and 8% of *Fusobacterium* species and 15 and 0% of *Clostridium* respectively [23]. In a European antibiotic susceptibility survey, conducted in 2000, 25/1284 *Bacteroides* strains were positive for c*f*iA carbapenem resistance gene and their study data exhibits a resistance rate of 0.8 and 1.3% for imipenem and meropenem respectively [9]. In a national survey conducted in Denmark, 3.9% of the *Bacteroides fragilis* isolates were resistant to meropenem whereas none of the isolates were resistant to metronidazole [24].

Metronidazole is one of the most commonly used antibiotic for the management of anaerobic infections especially *Bacteroides fragilis* infections. It is also being prescribed for prophylaxis, empirical and targeted therapy for many other infections in Pakistan. Although prior or prolonged metronidazole usage and development of resistance has been well reported for *Helicobacter* associated gastritis, studies reporting association of metronidazole overuse and emergence of resistance in anaerobic organisms are not available [25]. In vitro studies suggest that prolonged exposure of metronidazole to *nim* gene carrying anaerobes could lead to resistance [26]. A case control study evaluating risk factors for development of resistance against beta-lactam/beta-lactamase inhibitors in patients with *Bacteroides* bacteremia have identified duration of exposure as an independent risk factor of resistance [27]. Another study evaluating antibiotic exposure in past 2 years and isolation of resistant anaerobes from stool samples had shown an association with meropenem exposure and reduced susceptibility to meropenem. No association was found for metronidazole, clindamycin or piperacillin-tazobactam resistance [28]. In our study due to lack of reliable antibiotic usage data, we could not assess prior or prolonged use of metronidazole as a driver for resistance. Prolonged hospital stay was used as a surrogate for prolonged usage of metronidazole. Our findings suggest that prolonged hospital stay was not significantly associated with metronidazole resistance and probably not a good surrogate for prolonged antibiotic use. Further studies are required for assessment of relation of prolonged hospital stay and antibiotic usage to acquired resistance.

Imipenem is now widely used in hospital settings in case of complicated intra-abdominal infections. The previous data from Pakistan showed no resistance to carbapenems whereas our study shows 24.1% of imipenem resistance in metronidazole resistant strains, reflecting emerging resistance to carbapenems [12]. Imipenem resistance was also seen in at least one of the metronidazole susceptible isolates tested.

Our analysis showed that patients with malignancy or history of transplant had lesser chances of developing metronidazole resistance. Our study however lacked details on severity of disease, data of patient assessment using critical care scoring systems (APACHE II and SOFA scores) and antimicrobial therapy (single drug or combined regimen). A previous study conducted in our setup showed higher proportion of metronidazole resistance in patients with bacteremia [12]. However, this analysis showed that anaerobic strains isolated from cases of bacteremia had lower odds of metronidazole resistance.

The strength of this study is that this could be used as a baseline to guide future initiatives in implementing anaerobic susceptibility and subsequent generation of data of trends of antimicrobial resistance. There were however some major limitations of this study. This study was laboratory based study conducted in Southern Pakistan; hence an uneven distribution of isolates from other parts of the country was seen. The results therefore may not be generalizable to the whole country. Similarly, due to limited access to clinical data, association of other risk factors including prolonged antibiotic usage and detailed clinical status of cases could not be assessed. Finally, sample size for imipenem resistance; determined primarily in metronidazole resistant strains, was limited.

## Conclusion

In summary, an alarming 17.5% resistance amongst anaerobes against metronidazole is observed in this study. This resistance does not seem to be associated with bacteremia or mortality. However other risk factors like prior use of anti-anaerobic agents were not explored and may be important to evaluate in future. We also need to study the outcomes of antimicrobial resistance in anaerobes to understand its impact on individual patients and on public health. Due to emerging drug resistance in anaerobes, newer and alternative options for management also need to be explored. It is time that susceptibility testing of anaerobes should be a routine service, to guide physicians for proper management. This data may help in surveillance of resistance and in generating local antibiogram and can be used for periodic surveillance of resistance trend.

## Abbreviations

MIC: Minimum inhibitory concentration
M.I.C.E: MIC evaluator
CLSI: Clinical Laboratory Standards Institute
